# The effect of psychological distance on automatic goal contagion

**DOI:** 10.1080/23743603.2017.1288877

**Published:** 2017-03-21

**Authors:** Janet Wessler, Jochim Hansen

**Affiliations:** ^a^ Department of Psychology, University of Salzburg, Salzburg, Austria

**Keywords:** Construal-level theory, psychological distance, goal contagion, action identification, motivation

## Abstract

We investigated how psychological distance influences goal contagion (the extent to which people automatically adopt another person’s goals). On the basis of construal-level theory, we predicted people would be more prone to goal contagion when primed with psychological distance (vs. closeness) because they would construe the other person’s behavior in terms of its underlying goal. Alternatively, we predicted people primed with psychological closeness (vs. distance) would be more prone to goal contagion because closeness may increase the personal relevance of another’s goals – a process not mediated by construal level. In two preregistered studies, participants read about a student whose behavior implied either an academic or a social goal. We manipulated (a) participants’ level of mental construal with a mind-set task (Study 1) and (b) their social distance from another person who showed academic or social behaviors (Study 2). We measured performance on an anagram task as an indicator of academic goal contagion. For Study 1, we predicted that participants reading about academic (vs. social) behaviors would show a better anagram performance, especially when primed with an abstract mind-set. For Study 2, we predicted that construal level and relevance effects might cancel each other out, because distance triggers both high-level construal and less relevance. In contrast to the construal-level hypothesis, the mind-set manipulation did not affect goal contagion in Study 1. In accordance with the relevance hypothesis, psychological proximity *increased* goal contagion in Study 2. We discuss how the findings relate to previous findings on goal contagion and imitation.

Every day people read about and observe other people’s behavior (e.g. we see people running in the park). People might consciously decide to pursue the goals underlying the observed behavior (e.g. living healthily), but many times people quite unconsciously pursue goals that they inferred from the observation of others, a process called goal contagion (e.g. Aarts, Gollwitzer, & Hassin, 2004). Especially through the Internet, cell phones, and social media, people are constantly confronted with information about other people’s behavior. These others can be more or less psychologically distant from the observer (Trope & Liberman, 2010). For example, they can be close friends or merely acquaintances, live close by or far away. An open question is, however, whether the psychological distance from the other person enhances or decreases the extent to which people adopt the underlying goal of that other person’s behavior. On the one hand, one could hypothesize that with increasing distance, people will construe the behaviors of others more abstractly (Trope & Liberman, 2010), and hence will be better at inferring goals from concrete behavior; people might then be more likely to pursue the other’s goal themselves. On the other hand, with increasing distance, other people’s actions and thus their goals may become less personally relevant. People will then be less likely to pursue the other’s goal themselves.

In the current research, we explored the open question of what influence psychological distance and construal level have on goal contagion. To the best of our knowledge, this is the first research specifically designed to test the construal-level and relevance hypotheses against each other. The findings will broaden our understanding of the factors that affect social influence in general, and therefore have implications for applied settings.

Goal setting can be activated consciously (e.g. by explicitly instructing participants; Hamilton, Katz, & Leirer, 1980), but many times the process of setting and pursuing goals is influenced by situational cues that people are not directly aware of. These situational influences can include the priming of certain goals (Bargh, Gollwitzer, Lee-Chai, Barndollar, & Trötschel, 2001; Chartrand & Bargh, 1996) or unconscious contagion of the goals of other people (Aarts et al., 2004; Custers & Aarts, 2010). Bargh and colleagues (2001) showed that participants primed with a performance goal (vs. a control group) were more likely to continue with an interrupted task that satisfied this adopted goal. In a study by Aarts et al. (2004), participants read about another person who worked on a farm either voluntarily (control condition) or in exchange for money (which implied the goal of earning money). A message on a computer screen informed participants that they would next perform a mouse-click task and, if enough time was left, a final task in which they had the chance to earn money. As an indicator of striving toward the money-making goal, the researchers measured how quickly participants removed the message from the screen to get to the mouse-click task as well as their speed during the task. Participants in the money-making goal condition showed a goal contagion effect. That is, they were faster both in removing the screen and in the mouse-click task itself compared to participants in the control condition. Participants reported no awareness of being influenced. These results indicate that automatic goal pursuit can also occur just by reading something about another person’s behavior that implies a certain goal. The results relate to other empirical evidence showing that people automatically and unconsciously infer a goal that underlies an observed behavior (e.g. Hassin, Aarts, & Ferguson, 2005).

These and other findings suggest that the goal contagion effect depends on two processes (Aarts, Dijksterhuis, & Dik, 2008; Dik & Aarts, 2007): The perceiver must automatically infer a goal from the observed behavior, and the perceiver needs to pursue this goal with her or his own behavioral means. The first of these two processes is influenced by the level on which the observed behavior is construed. According to the action-identification theory (Vallacher & Wegner, 1987; Wegner & Vallacher, 1986), actions can be identified on a continuum from a low level (i.e. focus on action-related features) to a high level (i.e. focus on goal-related features). For example, a person may see another person reading a book in the library and construe this action either as flipping pages (low-level identification) or as studying for an exam (high-level identification). Generally, people prefer to identify actions on a high rather than low level of abstraction if both identification types are equally conceivable (Vallacher & Wegner, 1987). For example, Long and Golding (1993) found that when participants read a story that included actions that could be interpreted as having both superordinate and subordinate goals, the protagonist’s superordinate goal was more cognitively accessible afterward. Building on early studies by Heider and Simmel (1944) that demonstrated that people attribute mental states even to geometric shapes (for an overview, see Scholl & Tremoulet, 2000), later research has convincingly shown that people infer causes of overt behavior spontaneously and without conscious intent (Hassin, Bargh, & Uleman, 2002). Upon encoding a behavior, people draw spontaneous trait inferences (Uleman, Newman, & Moskowitz, 1996), but they also automatically infer intentions and goals of the actor (Hassin et al., 2005). For instance, participants in a study by Dik and Aarts (2007) saw an animated ball whose movements implied the goal of helping a smaller ball. The more effort the ball invested in trying to help, the more cognitively accessible was the helping goal afterwards (e.g. as measured with a lexical decision task), and the more pronounced was the effect on participants’ actual behavior, that is, the goal contagion effect (as measured by participants’ willingness to help). Summarizing, past research suggests that the goal contagion effect seems to rely on people’s automatic tendency to infer goals from observed actions.

Although the basic assumption of this line of research is that people automatically infer superordinate goals from quite concrete actions, there might be conditions that enhance spontaneous goal inference. In particular, construal-level theory (CLT; Trope & Liberman, 2010) assumes that psychological distance moderates one’s focus on low-level versus high-level action aspects. Behaviors of others can be more or less psychologically distant. For example, a person can watch a friend or a stranger perform a behavior (i.e. social distance), observe it in the here-and-now or watch it happen at a remote location in the past (i.e. spatial and temporal distance). CLT assumes that with increasing distance, people mentally represent behaviors (of others) on a higher level of abstraction and focus relatively more on goal-related aspects. For instance, participants perceived activities (e.g. studying) in terms of goals (doing well in school) instead of means (reading a textbook) when they imagined the activities in the distant (vs. near) future (Liberman & Trope, 1998) or at a spatially distant (vs. near) location (Fujita, Henderson, Eng, Trope, & Liberman, 2006). But no one has yet tested whether distance affects the contagion of goals. According to CLT, from a distance, people focus on higher level, goal-related aspects of actions. Therefore, we assume that high-level, goal-related aspects of others’ actions become more accessible from a distance, and people may be more likely to infer an underlying goal of an observed action of a distant versus close other. Consequently, our hypothesis derived from CLT is that people are more likely to adopt goals of distant (vs. near) others.

For the second goal contagion process, in which individuals automatically pursue an inferred goal, the goal must have an incentive value or be desirable for the individual (Aarts et al., 2008). In other words, the goal needs to be personally relevant. For instance, participants who were primed with social groups that are associated with helping behavior displayed more helping behaviors themselves, but this effect was enhanced for participants who already had a strong intrinsic helping motive (Aarts et al., 2005). Similarly, in the study described above in which participants read about another person who worked on a farm either voluntarily (control condition) or in exchange for money (which implied the goal of earning money), Aarts et al. (2004) measured the participants’ need for money. Those who were in high need of money showed a more pronounced goal contagion effect.

Interestingly, the subjective closeness of a goal might influence its relevance for the self and promote motivation and goal achievement (Peetz, Wilson, & Strahan, 2009). Participants who feel temporally closer to their goal performed better and were more motivated to work toward the goal than participants who felt subjectively distant (Peetz et al., 2009). Participants primed with a significant other person who had high expectations about the participants’ performance and highly valued the goal attainment positively influenced participants’ performance on an anagram task (Shah, 2003a). Moreover, previous research has found that both social (Loersch, Aarts, Payne, & Jefferis, 2008) and temporal (Leander & Shah, 2013) proximity promoted goal contagion. In one study by Loersch and colleagues (2008), participants watched a video in which two racquetball players were behaving either cooperatively or competitively. The video also displayed the players’ group membership: They were members either of the participants’ university or of another university. Afterward, participants imagined being a football coach in different scenarios and indicated the extent to which they would choose cooperative rather than competitive strategies. The goal contagion effect emerged only for participants who thought that the players were from their own university. In Leander and Shah’s (2013) research, participants imagined a friend with an academic deadline the next day or in the coming weeks. The academic achievement goal of the friend was more salient for participants if they had read about the temporally proximal deadline. This effect was even more pronounced when the friend’s deadline was relevant for the participants (i.e. the time frame of the friend’s deadline matched the time frame of the start of the participants’ assignment). Although these studies manipulated psychological distance, they disregarded the potential influence of level of construal on goal inferences in the contagion process because the goals were made relatively explicit. However, these previous findings still indicate that psychological closeness may increase the relevance of the observed behavior and thus the perceiver’s own acting upon the underlying goal (i.e. increase the goal contagion effect).

Summarizing, building on CLT, there are arguments for the assumption that goals of others’ actions may be relatively more salient from a distance, and thus the goal contagion effect would be more pronounced for distant others (Hypothesis A). On the other hand, there is reason to argue that goals of proximal others’ actions may become more relevant for oneself, and thus the goal contagion effect should be more pronounced for proximal others (Hypothesis B). In two studies, we tested the competing hypotheses against each other. Participants read a description of another student whose behavior was indicative of either an academic or a social goal. In Study 1, we manipulated participants’ level of construal with a mind-set prime, whereas Study 2 employed the manipulation of participants’ distance from the target. Afterward, participants engaged in an anagram-solving task that was intended to fulfill an academic performance goal. The dependent variable was the total number of correctly formed words from the anagrams, that is, the anagram performance. In Study 1, only the construal process could influence goal contagion. Here, we predicted a pattern consistent with Hypothesis A (see Figure 1): Participants primed with an abstract (“why”) mind-set would form more words in the anagram task compared to participants primed with a more concrete (“how”) mind-set if they had read vignettes of a student with an academic (vs. social) goal. In Study 2, both processes (construal and relevance) could occur, and either Hypothesis A, Hypotheses B, or both hypotheses could be valid (see Figure 1): participants primed with psychological distance would form either more words (Hypothesis A) or fewer words (Hypothesis B) in the anagram task compared to participants primed with psychological closeness if they had read vignettes of a student with an academic (vs. social) goal. If both hypotheses are true, the two predicted effects might cancel each other out, and we would predict no performance differences in the anagram task between participants primed with psychological distance and participants primed with psychological closeness. We began our exploration of these issues with two pre-registered pilot studies.

**Figure 1.**
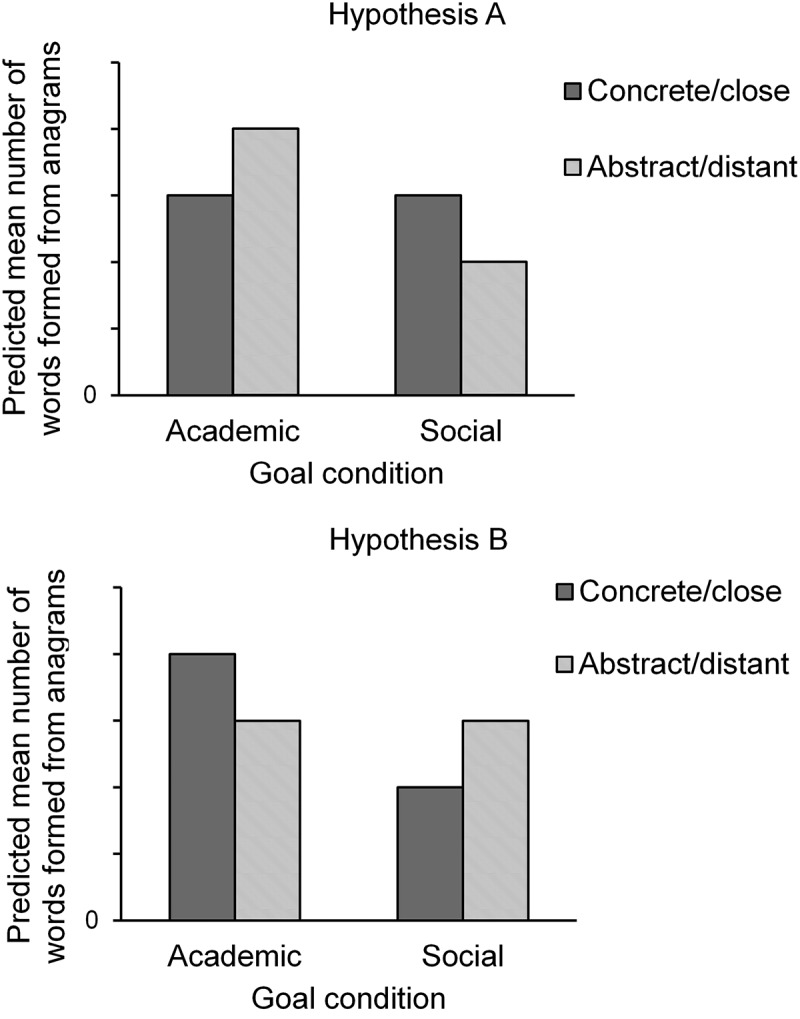
Predicted performance on the anagram task (mean number of words formed) if the construal-level hypothesis (upper panel) or the relevance hypothesis (lower panel) is valid.

## Pre-registered Pilot Study 1

For the current set of studies, we developed vignettes that describe the concrete behaviors of a target person with an implicit academic versus social goal. The action descriptions in the vignettes were based on the linguistic category model (Semin & Fiedler, 1988, 1991). This model distinguishes between four classes of words that vary from very concrete to very abstract verbal descriptions of actions. The most concrete words are descriptive action verbs that refer to specific behaviors in specific situations and describe an objective physical reality. Words belonging to this class are almost free from any element of interpretation (e.g. to write, to count). Interpretive action verbs are more abstract and leave more room for interpretation. They describe a whole class of behaviors, but they still refer to specific action episodes (e.g. to help, to insult). On the next level are state verbs that describe enduring states and cannot be observed (e.g. to hate, to love). Finally, adjectives are the most abstract word class. Rather than illustrating behavior they instead describe long-lasting characteristics. For the current studies, we developed vignettes that contained predominantly descriptive action verbs for two reasons. First, descriptive action verbs do not refer to any higher level goal and thus leave the task of inferring the goal from the actions to the participants themselves. Second, using descriptive action verbs enabled us to create two parallel versions of vignettes in which the construal level of actions was identical, but the content and implied goal of the actions differed. In this pilot study, we wanted to make sure that vignettes implying an academic or social goal were successful in increasing the cognitive salience of goal-related concepts measured with a categorization task as used by Leander and Shah (2013). We assumed that an academic achievement goal would be more salient for participants who read about actions implying an academic goal.

### Method

#### Sample-size calculation

We conducted an *a priori* sample-size analysis with G*Power 3.1.7 (Faul, Erdfelder, Lang, & Buchner, 2007). To estimate the size of the vignettes’ effect on performance of the categorization task, we used the results of Leander and Shah (2013, Study 1). With an Excel sheet provided by Lakens (2013), we estimated the size of the effect of the vignettes on the number of target words categorized as academic to be *ƞ*_p_^2^ = .16 (Cohen’s *d* = 0.88). With this effect size, an *a priori* power of 1 – *β* **= .80, an alpha error probability of *α* = .05, and two independent groups, the optimal sample size for the pilot study was 44 participants overall (22 per group). We planned to collect two additional participants per group, in case we had to exclude participants. This could be necessary if participants expressed suspicion regarding the connectedness of the two study parts (i.e. suspicions that the descriptions of the student influenced their performance in the categorization task). We probed this with a written funneled debriefing procedure (cf. Bargh & Chartrand, 2000). We planned to exclude from data analyses participants who reported any suspicions that the first part influenced their behavior in the second part (see questions 3 and 4 in Appendix A1). We also planned to exclude any participants who had outlying values on the number of target words identified as academic, with |*z*|≥ 3.29. Including four additional participants resulted in an overall sample size of 48. We report analyses with and without the excluded participants.

#### Participants and design

Overall, we tested 67 participants between 18 and 58 years of age (*M* = 23.88 years, *SD* = 6.35; 36 female, 31 male). As indicated by their answers to the funneled debriefing questions, a surprisingly large number of participants (23) were suspicious that the first study part influenced their behavior in the second study part. Because excluding all of these participants from the analysis would have resulted in a very low power, we replaced those participants and extended our planned sample size from 44 to 67 (as agreed upon with the editor of the journal) to get the optimal sample size according to the *a priori* sample-size calculation. Participants were randomly assigned to one of the two between-subjects goal conditions (academic vs. social). In the final sample without suspicious participants, we had 22 participants in the academic priming and 22 participants in the social priming condition.

#### Materials and procedure

We conducted Pilot Study 1 as preregistered. Participants were run in individual cubicles in a laboratory. After participants signed the informed consent form, the experimenter told them that they would first take part in a paper-and-pencil study about “getting to know other people” and then in an unrelated “decision-making” computer experiment. In the paper-and-pencil study, they read a vignette about another student called Anita and were asked to think about this person. In the academic goal condition, participants read the following:

The student Anita is really pleased with her studies and her university life. For example, she goes to the lectures and meets with fellow students in order to study together. She often sits in the library to read textbooks and articles and to write summaries. (Translated from German, see Appendix A2)

In the social goal condition, they read the following:

The student Anita is really pleased with her studies and her university life. For example, she goes to sports classes and meets with friends to chat. She often sits in a café to read magazines and social media updates and to write commentaries on the updates of her friends. (Translated from German, see Appendix A2)

On the next page, to increase the salience of the content and to create plausibility of the cover story (see Appendix A3), participants answered three multiple choice questions about the vignettes. After telling the experimenter that they had finished, participants started the ostensibly unrelated “decision-making” computer task. This word categorization task was used in previous research on goal contagion to measure cognitive goal salience (Leander & Shah, 2013; Leander, Shah, & Chartrand, 2011). After four practice trials, participants saw 30 words from three categories: social, academic, and ambiguous (translated into German from the original words used by Leander & Shah, 2013; see Appendix A4) in random order and decided by pressing one of two buttons if each word belonged to the category “social” or “academic.” Participants were instructed to make their decision as fast and accurately as possible. We predicted that those who read the academic goal vignettes would classify more of the ambiguous target words as belonging to the academic category. For exploratory reasons, we asked participants for their sex, age, and current grade point average (cf. Leander & Shah, 2013). Finally, participants completed a written funneled debriefing procedure (cf. Bargh & Chartrand, 2000, see Appendix A1). When we finished collecting data from the entire sample, interested participants were informed about the study background via e-mail.

### Results

#### Data preprocessing

The number of words the participants categorized was approximately normally distributed as indicated graphically by histograms and box plots. However, a significant Shapiro–Wilk test indicated that the assumption of normal distribution might be violated, *W*(44) = 0.94, *p* = .021. The variances in the two conditions were homogeneous, as indicated by a nonsignificant Levene test, *F*(1, 42) = 0.44, *p *= .51. There were no outliers in the sample, as *z* values ranged between – 2.58 and 2.21 (cf. Tabachnick & Fidell, 2013, Chapter 4).

#### Pre-registered analysis

A *t*-test on the number of ambiguous words categorized as academic did not reveal a significant difference between the two goal-priming groups, *t*(42) = 0.73, *p* = .471, *d* = 0.22. Participants in the social priming condition categorized as many ambiguous words (*M* = 4.46, *SD* = 1.68) as academic as participants in the academic priming condition (*M* = 4.09, *SD* = 1.63). When we included all participants in the analysis, there also was no significant difference between the groups, *t*(65) = 0.38, *p* = .70, *d* = 0.09. Because the dependent variable was not normally distributed, we additionally conducted a nonparametric Mann–Whitney *U*-test, which again indicated that there was no significant difference between priming conditions, *p* = .326.

#### Exploratory analyses

As a manipulation check of correct word categorization, we conducted a mixed 2 (goal priming: academic vs. social) × 3 (word category: academic vs. social vs. ambiguous) analysis of variance (ANOVA) with the number of words categorized as academic as the dependent variable. There was no main effect of goal priming, *F* < 1, but we did find a significant main effect of word category, *F*(2, 84) = 74.17, *p* < .001, *ƞ*_p_^2^ = .63. The number of academic words categorized as academic (*M* = 7.14, *SD* = 1.66) was significantly higher than the number of social words (*M* = 3.75, *SD* = 1.60) categorized as academic, *F*(1, 43) = 120.25, *p* < .001, *ƞ*_p_^2^ = .74, but social words and ambiguous words (*M* = 4.27, *SD* = 1.65) were equally often categorized as academic, *F*(1, 43) = 3.34, *p* = .075, *ƞ*_p_^2^ = .072. No interaction between goal priming and word category emerged, *F* < 1.

### Discussion

In Pilot Study 1, simply reading about another student showing academic versus social behaviors did not increase the salience of the academic goal, as indicated by the number of ambiguous words categorized as academic. This finding was not in line with our hypothesis, which stated that the vignettes should effectively prime an academic performance goal. There are three possible reasons for these findings. First, the experimental cover story might have prevented the priming from being successful: We explicitly told participants that they would participate in two different studies, one about “getting to know other people” and an unrelated “decision-making” study. Thus participants thought they had fully completed the first task before going on with the second, a condition that can even inhibit the accessibility of the primed goals (Liberman, Förster, & Higgins, 2007). Liberman et al. (2007) found that only participants who had been interrupted in a priming task showed an increased accessibility of prime-related concepts whereas participants who had completed a priming task showed inhibition of prime-related concepts. Considering these insights, we made a change in the experimental procedure in a second pilot study. Here, participants were told that they would work on two different tasks. After participants had worked on the first (goal-priming) task for 3 min, the experimenter interrupted them and asked them to start the second task (goal salience measure).

Second, the vignettes might not have been strong enough to induce an academic goal. Therefore, we changed the vignettes to make them more strongly suggestive of an academic or social goal. We modified the information about Anita by including more key words belonging to the academic (e.g. exam) or social (e.g. party) category (without explicitly mentioning a goal).

Also, in the funneled debriefing, some participants said that the multiple-choice questions were too easy, which made them suspicious. Therefore, we further obscured the purpose of the vignettes by making participants write a short text about the person in the vignettes (as we planned to do in Main Study 2). We expected this to additionally strengthen the goal-priming manipulation.

Third, although previous research established the word categorization task as a measure of goal salience after reading vignettes about another person (Leander & Shah, 2013; Leander et al., 2011), the measure might not have been sensitive enough to measure subtle differences between the priming groups. Therefore, we used an additional measure for goal activation, a lexical decision task (see Liberman et al., 2007).

## Pre-registered Pilot Study 2

#### Method Participants

Overall, we tested 51 participants (27 female, 24 male) between 18 and 31 years of age (*M* = 21.45 years, *SD* = 2.66), with 25 in the academic and 26 in the social priming condition. We had planned a sample size of 48 but collected data from an additional three participants, because three participants had not adhered to the experimenter’s instructions and started the goal salience measure before the priming task. The experimenter interrupted those participants, gave them the priming task, and they started the goal salience measure again. As they then performed the tasks in the correct order, we left those participants in the final sample (*N* = 51). In the funneled debriefing, 12 participants were suspicious that the first task influenced their behavior in the computer experiments. We report analyses with and without those participants.

#### Procedure

As in Pilot Study 1, participants were tested in individual cubicles in the laboratory. After signing an informed consent form, they received extended vignettes about the student Anita. The new vignettes for the academic goal condition read as follows:

The student Anita is really pleased with her studies and her university life. For example, she goes to the lectures and takes notes about the content. Also she meets with fellow students in the canteen to study together for the exams and to discuss. She often sits in the library to read textbooks and articles and to write summaries. She informs herself about talks or discussions in her neighborhood and likes to attend them very much. In her room in the dormitory she has a collection of scientific journals on her shelf and she converses with her roommates about the topics of her university courses. (Translated from German, see Appendix A5)

The new vignettes for the social goal condition read as follows:

The student Anita is really pleased with her studies and her university life. For example, she goes to sports classes and meets with friends to chat. Also she meets with fellow students in the canteen to drink coffee together and to talk. She often sits in a café to read magazines and social media updates and to write commentaries on the updates of her friends. She informs herself about parties or flea markets in her neighborhood and likes to go to them very much. In her shared room she has a collection of pop CDs on her shelf and she exchanges news of their common friends with her roommates. (Translated from German, see Appendix A5)

Afterward, this time participants were asked to write down five full sentences about what they thought Anita was like (with the prompt “Imagine, for instance, what she is like, what she feels and thinks, and/or how she behaves”). After 3 min, the experimenter asked participants to start with the first computer task, which was the lexical decision task to measure goal salience. In this task, participants were presented with 120 letter strings and had to decide as fast and accurately as possible whether these were German words or nonwords by pressing one of two buttons. Of the 60 words (see Appendix A6), 20 belonged to the category “social,” 20 to the category “academic,” and 20 to no specific category (“control”). After this first task, the experimenter started the second goal salience task, which was the word categorization task used in Pilot Study 1. Participants then received a final questionnaire that included three multiple-choice items about Anita (as used in Pilot Study 1), a place to report their demographics (sex, age, major, and current grade point average), and the written funneled debriefing.

### Results

#### Data preprocessing lexical decision task

We excluded reaction times for incorrect answers in the word trials (1.9% of all words trials; cf. Bargh, Chaiken, Govender, & Pratto, 1992). Inspection of the mean reaction times to social, *W*(51) = 0.88, *p* < .001, academic, *W*(51) = 0. 87, *p* < .001, and control, *W*(51) = 0. 86, *p* < .001, words revealed that they were not normally distributed. Therefore, we log-transformed the respective means (cf. Fazio, 1990). This led to graphical improvements in the distributions, but still a significant Shapiro–Wilk test indicated a violation of normality [social words: *W*(51) = 0. 94, *p* = .016; academic words: *W*(51) = 0. 95, *p* = .028; control words: *W*(51) = 0. 97, *p* = .010]. Variances between the social and academic priming groups were equal for all three word categories, *F*s < 1. Looking at the *z* values, no outliers were found (*z* between – 1.74 and 3.05).

#### Pre-registered analysis

**Figure 2.**
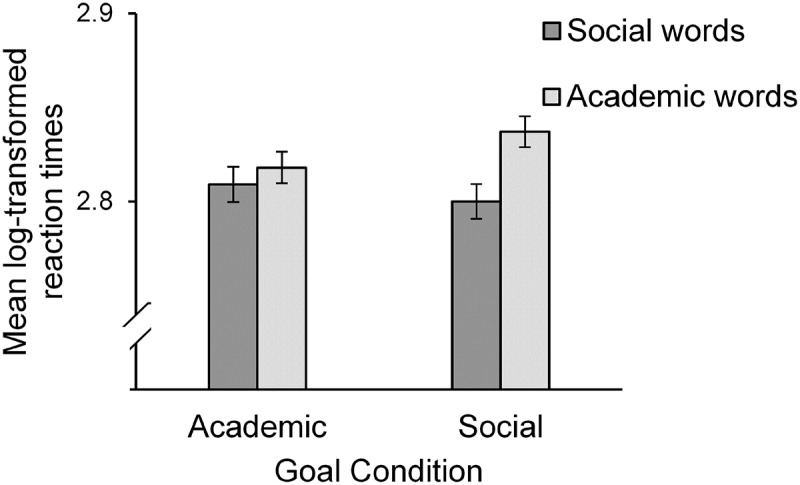
Mean log-transformed reaction times to words in the social category versus words in the academic category depending on social versus academic priming condition. The mean log-transformed reaction time for control words served as the covariate. Error bars represent ± 1 *SE.*

A 2 (priming condition: social vs. academic) × 2 (word category: social vs. academic) analysis of covariance (ANCOVA) on the log reaction time for social and academic words with the log reaction time for control words as a covariate revealed a significant interaction between priming condition and word category, *F*(1, 48) = 4.23, *p *= .045, *ƞ*_p_^2^ = .08. No other effects were significant, *F*s < 1. Bonferroni-adjusted simple comparisons showed that participants in the social priming condition reacted faster to words belonging to the social category (*M* = 2.80, *SD* = 0.01) than to words belonging to the academic category (*M* = 2.84, *SD* = 0.01), *F*(1, 48) = 14.62, *p* < .001, *ƞ*_p_^2^ = .23. In the academic priming condition, there was no difference in reaction times to words belonging to the social category (*M* = 2.81, *SD* = 0.01) and words belonging to the academic category (*M* = 2.82, *SD* = 0.01), *F*(1, 48) = 0.67, *p* = 42, *ƞ*_p_^2^ = .01. Results are shown in Figure 2.

We conducted the same analysis without suspicious participants (leaving 39 in the analysis: 23 in social, 16 in academic). A 2 (priming condition: social vs. academic) × 2 (word category: social vs. academic) ANCOVA on the log reaction time for social and academic words with the log reaction time for control words as a covariate revealed no significant effects, *F*s < 2.17.

#### Data preprocessing word categorization task

Graphical inspections and statistical tests revealed that the number of ambiguous words categorized as academic were not normally distributed, *W*(51) = 0.94, *p* = .015. Also, the variances in the two groups were not equal, as indicated by a significant Levene test, *F*(1, 49) = 4.20, *p* = .046. Looking at the *z* values, no outliers were found (*z* between – 1.80 and 1.91). Because of the nonnormality of our data, we ran a nonparametric test of the difference of the means in addition to the independent samples *t*-test.

#### Pre-registered analysis

An independent samples *t*-test revealed that participants who had read about Anita being social categorized as many ambiguous words as academic (*M* = 4.23, *SD* = 1.68) as participants who had read about Anita being academic (*M* = 4.56, *SD* = 2.10), *t*(45.91) = −0.62, *p* = .54, *d* = 0.17. Also, a Mann–Whitney *U*-test for independent samples showed that the distribution of words categorized as academic did not differ between the two priming groups, *p* = .44.

For the sample without suspicious participants, an independent samples *t*-test on the sum of ambiguous words categorized as academic revealed no difference between the group that read about Anita being social (*M* = 4.13, *SD* = 1.68) and the group that read about Anita being academic (*M* = 4.38, *SD* = 2.09), *t*(27.75) = −0.39, *p *= .70, *d* = 0.13. A Mann–Whitney *U*-test for independent samples showed that the distribution of words categorized as academic did not differ between the two priming groups, *p* = .703.

#### Exploratory analysis

Because we conducted the word categorization task in Pilot Studies 1 and 2, we were able to analyze the data across both samples. Moreover, this procedure allowed us to include sex as a potential moderator of the effect, because (although not originally planned) we had collected data of both men and women in the pilot studies. A 2 (sex: female vs. male) × 2 (study: Pilot Study 1 vs. Pilot Study 2) × 2 (goal priming: social vs. academic) between-subjects ANOVA on the number of words categorized as academic revealed no significant main effects or interactions, all *F*s < 2.29. Similarly, an independent samples *t*-test for female participants only (29 in social, 33 in academic goal-priming condition) showed no significant difference between the social (*M* = 4.41, *SD* = 1.21) and the academic (*M* = 4.55, *SD* = 1.84) condition, *t*(55.83) = –.34, *p* = .738, *d* = 0.09.

### Discussion

The aim of Pilot Study 2 was to improve the vignettes that were intended to prime a social versus academic goal. Additionally, we aimed at including a more sensitive measure of cognitive goal accessibility. In the lexical decision task, we found differences in reaction times for social versus academic words after the social versus academic priming. Participants who had read the social vignettes responded more slowly to words in the academic category, indicating that the competitive goal of social activities was suppressed in these participants. Overall then, the vignettes seem to have effectively primed a social versus academic goal.

Replicating the findings of Pilot Study 1, in the word categorization task participants showed no increase in salience of the academic concept after having read the academic goal vignettes. Although this task has been used in the goal contagion literature (Leander & Shah, 2013), in our studies we were not able to detect a goal-priming effect on this measure. One major reason might be that we based our sample-size calculations on the effect size of *d* = 0.88, a large effect reported in previous research with the word categorization task (Leander & Shah, 2013). In our studies, however, we found only very small effect sizes of *d* = 0.22 (Pilot Study 1) and *d *= 0.17 (Pilot Study 2). The resulting achieved power with our preplanned sample size therefore was too low for us to have detected any possible existing effects (11.2% in Pilot Study 1 and 9.32% in Pilot Study 2). In contrast, the reaction times in the lexical decision task seemed to be more sensitive to capturing the small effects of goal priming in our study, with an achieved power of 54.13%.

In both pilot studies, we were not able to find priming effects when we excluded suspicious participants from the studies, probably because this resulted in an even lower achieved power. For example, the achieved power of the lexical decision task in Pilot Study 2 dropped from 54.13% to 32.10% when we excluded the 12 suspicious participants. Usually, an effect of suspicions on the reaction times would not be expected, because it can be difficult to consciously manipulate reaction times. Therefore, the nonsignificant findings for the smaller sample with only nonsuspicious participants were probably due to the low power of the analyses.

Taking the results of the two pilot studies into consideration, we made some slight changes in the preregistered method of the main studies. First, in Study 1 we used the vignette priming procedure that we had planned to use for Study 2 only but which we had now successfully applied in Pilot Study 2: In both Studies 1 and 2, participants read the extended versions of the Anita vignette and wrote a short text about her for 3 min. Second, especially the psychology students had been suspicious that reading the vignettes influenced their behavior in the goal salience tasks. We thus decided for the main studies to collect data not only from psychology students but also from students of various majors. However, our preregistered social distance manipulation in Study 2 had been related to Anita’s academic major (psychology vs. political science). We hence changed the psychological distance manipulation to presenting Anita as a student at the University of Salzburg (socially close) versus at the University of Vienna (socially distant), without giving information about her academic major.

## Pre-registered Study 1

The pilot studies served to establish the influence of the vignettes on goal salience. In this first main study we manipulated participants’ level of construal with a how–why mind-set prime (cf. Alter, Oppenheimer, & Zemla, 2010; Freitas, Gollwitzer, & Trope, 2004) before participants read the vignettes. Afterward, we measured if participants themselves pursued the implied goal in a different unrelated task, which has been used as a measure of academic goal contagion before (solving anagrams; e.g. Leander & Shah, 2013). We predicted that a high level of construal (i.e. a “why” mind-set) would help participants infer the underlying cause of the target person’s behavior and therefore her goal (Dik & Aarts, 2007). This enhanced goal inference might consequently result in higher goal pursuit in their own behavior (see Dik & Aarts, 2007). Moreover, a higher level of construal should enable the participants to use new goal-related behaviors that differed from the observed behavior while following the same goal as the target person (Hansen, Alves, & Trope, 2016).

### Method

#### Sample-size calculation

We conducted an *a priori* sample-size analysis with G*Power 3.1.7 (Faul et al., 2007). To estimate the goal contagion main effect, we relied on the effect size of *ƞ*_p_^2^ = .036 (*d* = 0.386) as calculated from the goal contagion main effect results of Aarts et al. (2004, Study 1). Please note that studies looking at interaction effects between goal vignettes and another experimentally manipulated variable (e.g. group affiliation of the target) have reported even higher effects (e.g. *ƞ*_p_^2^ = .053 [*d* = 0.473]; Loersch et al., 2008, Study 2). We based our estimate on the more conservative small effect size. This estimate is also in line with a recent meta-analytical estimate of *d* = 0.33 for (goal) priming effects in general (Weingarten et al., 2016).

Based on the effect size of *ƞ*_p_^2^ = .036, an *a priori* power of 1 – *β* **= .80, an alpha error probability of *α* = .05, and two independent groups, the optimal sample size for Study 1 was 213 participants overall (53.25 in each of four groups). We planned to collect two additional participants per group in anticipation of possible exclusions. First, we planned to replace participants who expressed suspicions regarding the connectedness of the three study parts (i.e. suspicions that the mind-set task or the descriptions of the student influenced their performance in the anagram task). We probed suspicions with a written funneled debriefing procedure (cf. Bargh & Chartrand, 2000; see question 3 and 4 in Appendix B1). Second, we planned to replace participants who had outlying values on the number of target words identified as academic, with |*z*|≥ 3.29. This results in an overall planned sample size of 220 (55 per group).

#### Participants and design

We recruited German-speaking female students from diverse social online networks and the participant panel Prolific Academic. Overall, 393 participants opened the study link, of which 323 (82.19%) agreed to the informed consent and indicated being female, a student, and having German as a mother tongue. They were randomly assigned to one of the four experimental conditions of the 2 (mind-set: how vs. why) × 2 (goal: academic vs. social) between-subjects design. Of these participants, 301 started the how–why task (76.59%; 137 how, 164 why), during which 65 participants (21.59%) dropped out. Of the remaining participants, 117 read the academic vignettes about Anita (58 in the why, 59 in the how condition), and 119 read the social vignettes (67 in the why, 52 in the how condition). Overall, 228 participants started the anagram task, of which 221 finished it. At the end, 219 participants indicated their demographics and responded to the funneled debriefing. Our final sample included all participants who had completed the anagrams (*N* = 221; 54 in why-academic, 64 in why-social, 53 in how-academic, 50 in how-social). This deviated slightly from our planned sample size of 55 in each condition because of the dropouts. All participants were female, students of different majors, and between 18 and 51 years of age (*M* = 24.03 years, *SD* = 4.50). Of the 221 participants, 13 reported suspicions that the study was a priming study and that the description of Anita might have influenced their behavior in the anagram task. We report analyses with and without these participants.

#### Procedure

On a welcome page, participants were told that they would work on three different unrelated tasks, an imagination task, a task about getting to know other people, and an academic performance test. After giving informed consent, participants started with the imagination task (i.e. the mind-set manipulation) in which they received a list of six activities (cf. Alter et al., 2010). Under each activity, there was a box that was connected with an arrow to the activity. For half of the participants this arrow pointed down on the box, asking them to write down “how” they performed the activity (see Appendix B2). For the other half the arrow pointed up from the box to the activity, asking them to write down “why” they performed the activity (see Appendix B3). When finished, participants read the same vignettes about Anita as in Pilot Study 2 and were asked to write five sentences about Anita in the next 3 min (participants could continue with the study only after 3 min). After 5 min, the screen automatically switched to the next study page.

Next, participants worked on the academic performance test. The material informed participants that this study was about finding out how the vocabulary knowledge of people is associated with their university success, as previous research had (ostensibly) shown that performance in this test is positively related to students’ overall academic success. They were informed that individuals with high academic performance can find 80% of possible words in the anagram task (cf. Shah, 2003a). This information was given to ensure that the task was construed as being related to academic performance. Participants then engaged in an anagram task as used in previous goal contagion research (cf. Leander & Shah, 2013; Leander et al., 2011). They received six trials of five-letter strings (e.g. NGLAE; see Appendix B4 and formed as many five-letter German words as possible from the letters (e.g. Angel, Nagel, Algen). Each anagram had a minimum of five possible solutions. Participants’ performance score was the total number of correctly formed words. Previous research (Leander & Shah, 2013; Shah, 2003a) additionally used anagram persistence (in seconds) as a measure of goal contagion. However, they found very similar results for the performance and the persistence measure. Thus we refrained from additionally measuring anagram persistence.

At the end, we had participants answer the three multiple choice items about Anita as in the pilot studies in order to underline the cover story. They then reported their age, sex, academic major, semester, and their current university grade point average. They completed a written funneled debriefing procedure (cf. Bargh & Chartrand, 2000) that asked them about any suspicions regarding the connectedness of the three study parts and the purpose of the study (see Appendix B1). They could leave their e-mail address if they were interested in receiving information about the study background after data collection for the total sample was finished.

### Results

#### Data preprocessing

The number of correctly formed anagrams was graphically and statistically normally distributed as indicated by histograms and nonsignificant Shapiro–Wilk tests in all conditions (*p*s > .058) except the why-social condition, *W*(64) = 0.94, *p* = .002. The variances in the four conditions were homogeneous, as indicated by a nonsignificant Levene test, *F*(3, 217) = 0.85, *p *= .469. There were no outliers in the sample, as *z* values ranged between – 1.98 and 2.99.

#### Preregistered analysis

A 2 (mind-set: how vs. why) × 2 (goal: academic vs. social) between-subjects ANOVA with number of correctly generated words as the dependent variable revealed no main effect of mind-set, *F < *1.38, *p* = .241, *ƞ*_p_^2^ = .01, nor an interaction between mind-set and goal, *F*(1, 217) = 0.21, *p* = .884, *ƞ*_p_^2^ < .001. Participants in the how mind-set conditions who were primed with the academic (*M* = 13.23, *SD* = 4.99) versus social (*M* = 14.18, *SD* = 4.94) behavior solved as many anagrams as participants in the why mind-set conditions who were primed with the academic (*M* = 13.74, *SD* = 5.56) versus social (*M* = 14.48, *SD* = 5.71) behavior. An analysis without participants who reported suspicions also showed no main effect of mind-set, *F*(1, 217) = 0.11, *p* = .918, *ƞ*_p_^2^ < .001, no main effect of goal, *F*(1, 217) = 1.99, *p* = .160, *ƞ*_p_^2^ = .01, and no interaction between mind-set and goal, *F*(1, 217) = 0.017, *p* = .896, *ƞ*_p_^2^ < .001.

#### Exploratory analyses

Because this study was conducted online, we wanted to make sure that all participants had adhered to the instructions of the how–why priming task. As a manipulation check, an independent judge blind to the experimental conditions coded 221 responses to the six items of the how–why task (see Fujita, Trope, Liberman, & Levin-Sagi, 2006; Hansen & Trope, 2013; Liberman & Trope, 1998; for a similar procedure). If a response reflected a subordinate means to the respective action, the judge coded the response with – 1, and if it reflected a superordinate end to the action the judge coded it as +1. If a participant’s response fit neither criterion, the judge coded it as 0. The values of each participant’s six responses were summed to create an index of level of construal with a potential range of – 6 to +6. Participants responses to the how-items were more related to the subordinate means (*M* = −5.74, *SD* = 0.78) than the responses to the why items (*M* = 5.72, *SD* = 0.98), as indicated by an independent samples *t*-test, *t*(219) = −95.41, *p* < .001, *d* = 12.94. In line with previous laboratory research (Fujita et al., 2006; Hansen & Trope, 2013; Liberman & Trope, 1998), the construal-level manipulation with the how–why task was successful in our online study.

### Discussion

In Study 1, we tested the hypothesis that a high-level construal might facilitate the process of inferring an underlying goal when observing a behavior of another person. However, we found no evidence in favor of the construal-level hypothesis: Participants who were primed with a low-level mind-set (“how”) formed as many correct anagrams as participants with a high-level mind-set (“why”). One possible explanation of this finding might be that – although construal level enhanced the process of inferring a goal – the motivation to pursue a stranger’s goal for oneself might be lacking (Shah, 2003a). This assumption is also in line with the finding that participants who had read about a student with an academic goal generally did not perform better in the anagram task than those who had read about a student with a social goal. Reading about an unknown student may have activated the goal but not necessarily motivated participants themselves to pursue this goal. As Fitzsimons and Fishbach (2010) argued, merely activating goals is not a sufficient condition for goals to influence psychological processes. This influence also depends on the goal’s “motivational priority.” Therefore, in Study 2, we aimed at manipulating the cognitive mental level of construal as well as the potential self-relevance of the other’s goal by attaching psychological distance to the model’s actions themselves (Trope & Liberman, 2010). This enabled us to directly test the cognitive construal-level hypothesis against the motivational relevance hypothesis.

## Pre-registered Study 2

In Study 2, we manipulated social distance from the described target instead of participants’ mind-sets. We recruited students of various academic majors (including but not limited to psychology students) because psychology students were most suspicious of the priming manipulation in the pilot studies. We, therefore, changed the preregistered social distance manipulation (i.e. psychology vs. a different major) in a way that we were able to test students from various majors. In particular, we stated that Anita was a student at the University of Salzburg versus the University of Vienna. This manipulation possibly added a relevance component to the construal of the observed behavior. We explored whether the construal effect (Hypothesis A) or the relevance effect (Hypothesis B) dominated.

### Method

#### Sample-size calculation

We conducted an *a priori* sample-size analysis with G*Power 3.1.7 (Faul et al., 2007). On the basis of a recent meta-analysis on the effect of psychological distance on diverse downstream consequences (Soderberg, Callahan, Kochersberger, Amit, & Ledgerwood, 2015), one can assume a medium-sized effect of Hedges’s *g* = 0.526 (*f* = 0.26) for the influence of distance on goal contagion. However, as outlined above, the goal contagion effect itself can be assumed to be rather small. Therefore we based our effect size estimate on the more conservative goal contagion effect estimate of *η* ^2^_p_ = .036 (*d* = 0.386). We applied the same criteria to exclude participants as in Study 1, with the addition of exclusion of participants who failed to reproduce the university affiliation of the target (which we used to manipulate social distance), because we could not be sure that the social distance manipulation was successful for these participants. As explained in Study 1, this results in an overall sample size of 220 (55 participants per group).

#### Participants and design

We recruited 220 female undergraduate students between 17 and 60 years of age (*M* = 21.98 years, *SD* = 4.47) at the University of Salzburg to participate in the study for course credit or financial reward (4 euros). They were randomly assigned to one of the four experimental conditions of the 2 (distance: close vs. distant) × 2 (goal: academic vs. social) between-subjects design, with 55 participants in each of the four conditions.

#### Materials and procedure

Participants were run in groups of one to six per session. Each participant received an informed consent form, a verbal description task (containing the distance manipulation, the description of another student called Anita, and an imagination task regarding meeting Anita), and an academic performance test.

After participants signed the consent form, the experimenter asked them to work on the verbal description task for 3 min. Participants imagined meeting Anita (“Imagine that you are meeting Anita, another student, for the first time. Both of you start chatting about university life. She tells you that …”). To manipulate social distance from Anita, she was introduced as an undergraduate student from the University of Salzburg (i.e. socially close) or the University of Vienna (i.e. socially distant). Participants wrote down five full sentences about what they imagined Anita to be like (along with the prompt “Imagine, for instance, what she is like, what she feels and thinks, and/or how she behaves). After 3 min, the experimenter asked participants to start the academic performance task. The materials and procedure were the same as described in Study 1.

After the academic performance test, participants were asked to answer the three multiple choice items used in the previous studies and additionally to recall Anita’s university in the verbal description task with an open question. As mentioned above, we analyzed only the data of participants who correctly remembered Anita’s university.

When finished, participants answered a final questionnaire that included three items measuring how relevant the academic performance goal in general was to them. For two of these items, participants indicated on a scale from 1 (*not at all*) to 7 (*very much*) how important their studies were to them (i.e. “How important is it for you to finish your studies with an ‘excellent’ average grade?” and “How important is it for you to be better than other students in your course?”). One bipolar item asked participants how important their studies were to them in relation to their leisure time on a scale from 1 (*leisure time more important*) to 7 (*studies more important*). Also, they reported their age, sex, academic major, semester, and their current university grade point average. Finally, participants completed a written funneled debriefing procedure (cf. Bargh & Chartrand, 2000) that asked them about any suspicions regarding the connectedness of the two study parts and the purpose of the study. Participants who reported any suspicions that the first part of the study influenced their behavior in the second part were excluded from data analyses. Once we had collected the data of the overall sample, interested participants were informed about the study background via e-mail.

### Results

#### Inclusion criteria

Of the 220 participants, 12 reported suspicions that the description of Anita might have influenced their behavior in the anagram task. We report analyses with and without these participants. However, 31 participants were not able to freely recall the distance manipulation correctly. We excluded these participants from data analysis.

#### Construal-level scores

Participants’ written descriptions of the meeting with Anita were analyzed with the linguistic category model (e.g. Semin & Fiedler, 1988). Two coders independently determined the abstractness level of each of the five sentences that participants wrote about Anita by performing the following steps. First, following the linguistic category model, they divided each sentence into its basic units (i.e. into part-sentences that each included a subject and an action, and for which one of the four word classes could be identified). Then the coders scored each basic unit for including a descriptive action verb (1), an interpretive action verb (2), a state verb (3), or an adjective (4). In this way, all units were coded for their abstractness on a numerical scale from 1 to 4. These scores were averaged for all units to measure the overall degree of abstractness of the description. This score significantly correlated between the two coders, *r* = .593, *p* < .001, but was slightly lower than the predefined cut-off of *r* > .60. Therefore, the coders discussed six ambiguous cases for which their overall abstraction scores differed by more than 1.5 units. Afterward, the abstraction scores correlated satisfactorily, *r* = .687, *p* < .001, and were averaged into a single index of construal level on which participants represented Anita. As a manipulation check, we tested with an independent samples *t*-test if this index differed between the two distance conditions. Participants who thought Anita was a student at the University of Vienna did not describe Anita more abstractly (*M* = 2.91, *SD* = 0.41) than those who thought she was at the University of Salzburg (*M* = 2.84, *SD* = 0.48), *t*(177) = −1.03, *p* = .306, *d* = 0.16.

#### Data preprocessing

The number of correctly generated words was normally distributed in each experimental condition, all *p*s > .172 in Shapiro–Wilk tests. Also, the variances in the four groups were homogeneous, *F*(3, 175) = 1.28, *p* = .283. There were no outliers; *z* values ranged from – 2.53 to 2.30. The reliability of the academic relevance score consisting of the three relevance items was not satisfactory (Cronbach’s *α* = .576). Examination of the correlations revealed that only the items “How important is it for you to finish your studies with an ‘excellent’ average grade?” and “How important is it for you to be better than other students in your course?” correlated satisfactorily, *r *= .61, *p* < .001. Therefore, we calculated the academic relevance score with these two items. An independent samples *t*-test as a manipulation check showed that participants in the socially close condition reported similar levels of academic goal relevance (*M* = 3.84, *SD* = 1.55) to that of participants in the socially distant condition (*M* = 3.87, *SD* = 1.54), *t*(177) = −0.11, *p* = .914, *d* = 0.01. The correlations between the study variables (i.e. distance condition, abstractness scores, relevance scores, and anagram performance) were all nonsignificant, all |*r*|< .12.

#### Pre-registered analysis

A 2 (distance: close vs. distant) × 2 (goal: academic vs. social) between-subjects ANOVA with number of correctly generated words as the dependent variable revealed no main effect of distance or of goal, *F*s < 1. However, an interaction between mind-set and goal emerged, *F*(1, 175) = 4.96, *p* = .027, *ƞ*_p_^2^ = .028 (achieved power: 61.72%). Bonferroni *post hoc* tests showed that participants who were spatially close to Anita solved marginally significantly more anagrams when they had read academic vignettes (*M* = 15.26, *SD* = 4.54) than when they had read social vignettes (*M* = 13.33, *SD* = 5.31), *F*(1, 175) = 3.18, *p* = .076, *ƞ*_p_^2^ = .018. Participants who were distant from Anita solved as many anagrams when they had read social vignettes (*M* = 15.21, *SD* = 4.14) as when they had read academic vignettes (*M* = 13.92, *SD* = 5.11), *F*(1, 175) = 1.80, *p* = .18, *ƞ*_p_^2^ = .01. Participants who had been primed with a social goal solved marginally fewer anagrams in the psychologically close versus distant condition, *F*(1, 175) = 3.36, *p* = .068, *ƞ*_p_^2^ = .019. For the sample without suspicious participants, a 2 (distance: close vs. distant) × 2 (goal: academic vs. social) between-subjects ANOVA with number of correctly generated words as the dependent variable revealed no main effect of distance or of goal, *F*s < 1. The interaction between mind-set and goal now was marginally significant, *F*(1, 164) = 3.52, *p* = .062, *ƞ*_p_^2^ = .021, but the achieved power of this analysis dropped to 47.10% due to the smaller sample size.

**Figure 3.**
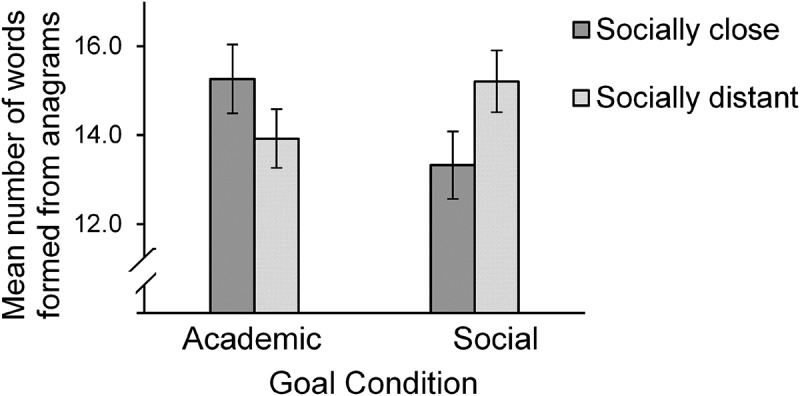
Mean number of correctly formed anagrams in Study 2 as a function of the social distance from the target person. Error bars represent ± 1 *SE.*

As shown in Figure 3, the pattern of the interaction is consistent with the relevance hypothesis, which assumes that psychological closeness to the model increases the goal contagion effect (Hypothesis B). Participants in the academic goal condition showed a better performance in the anagram task when Anita was a student at the University of Salzburg (socially close) versus at the University of Vienna (socially distant), because Anita’s behavior might have been more relevant to them. Participants in the social goal condition, in contrast, showed a lower performance when they were psychologically close to Anita, because they presumably focused on the social goal, which conflicted with an academic goal.

#### Exploratory analysis

Although we found an interaction pattern supportive of the relevance hypothesis (Hypothesis B), we still tested if including the construal level or the relevance scores, respectively, as covariates in the analysis would change this pattern. The 2 (distance: next day vs. in one year) × 2 (goal: academic vs. social) between-subjects ANCOVAs with the construal level score of the model’s actions as the covariate again revealed a significant interaction between mind-set and goal, *F*(1, 174) = 4.99, *p* = .027, *ƞ*_p_^2^ = .028. No main effects emerged, all *F*s < 1. An ANCOVA with the general relevance of an academic goal as the covariate also revealed a significant interaction between mind-set and goal, *F*(1, 174) = 4.87, *p* = .029, *ƞ*_p_^2^ = .027.

### Discussion

In Study 2, participants who were socially close to the other student showed an anagram performance that indicates they had adopted the underlying goal of the other student. If they had read about a student’s behavior with an academic (vs. social) goal, participants solved more (fewer) anagrams in a task that was presented as an academic performance test. This pattern of results is consistent with the hypothesis that psychological closeness promotes the relevance of the other’s goal for oneself and hence increases goal adoption (Hypotheses B). However, participants did not report higher levels of general academic goal relevance when they were socially close; controlling for general academic goal relevance did not change the pattern of results. The self-report measure of general relevance might not have caught the motivational mechanisms underlying the goal contagion effect.

We argued that psychological distance could increase goal adoption because a higher level of construal facilitates goal inference (Hypothesis A). However, as in Study 1 in which we had manipulated level of construal directly, we found no support for this hypothesis in Study 2, even if we used measured construal level as predictor. One explanation for this null effect might be that the goal inference process is very quick and automatic (Hassin et al., 2005, 2002), and therefore is very difficult to influence with a situational variable such as level of construal. Also, although a high level of construal enhanced the goal inference process, participants might not have applied the goal to their own behavior, due to a decreased motivational self-relevance associated with psychological distance. Critically, however, the social distance manipulation did not affect the abstractness of descriptions about the other student, in contrast to previous findings on descriptions of psychologically distant others (e.g. Fujita et al., 2006). Our manipulation might thus not have elicited a low versus high level of construal. However, even though the manipulation checks were nonsignificant, the social distance manipulation still influenced participants’ performance on the anagram task. Social distance therefore seemed to change the relevance of the target person’s behavior rather than the construal level of the behavior.

## General discussion

In this preregistered line of research, we tested to what extent the automatic transfer of goals from one person to another depends on the situationally induced level of construal and psychological distance between the two persons. On the one hand, we had hypothesized that goal contagion effects might be enhanced by a high level of construal, because representing the actions of the other person in terms of that person’s underlying goals (“why” the person conducts the action) might facilitate the process of adopting the same goal for oneself (Aarts et al., 2004; Dik & Aarts, 2007; Hassin et al., 2005; Trope & Liberman, 2010; Vallacher & Wegner, 1987). On the other hand, we had hypothesized that psychological closeness to the other person might promote the relevance of the other’s goal for oneself (Leander & Shah, 2013).

In Study 1 (which tested Hypothesis A), we did not find evidence for the idea that construal level influences goal contagion. Priming participants with a low-level “how” mind-set versus a high-level “why” mind-set (as done in previous research; Alter et al., 2010) did not influence the extent to which participants adopted an academic performance goal of another student. In Study 2, Hypotheses A and B were tested against each other: by manipulating psychological distance, both the construal level and the relevance process could potentially exert their effects on goal contagion. The findings provide support for Hypothesis B: After reading about a student with an academic (vs. social) goal, participants performed better (worse) on an anagram task if the other student was from their home university rather than a socially distant university. It thus seemed that the motivational relevance component underlying the goal contagion effect was more critical for goal contagion to occur than level of construal.

The current results replicated previous findings showing that goal contagion is stronger for socially close rather than distant others (Loersch et al., 2008). Like in our study, Loersch et al. (2008) manipulated social distance from target persons via university affiliation. Participants watched videos of cooperative versus competitive ball players. In the video footage, participants saw “Ohio State University” in the socially close and “University of Toronto” in the socially distant condition. Participants reported higher competitiveness after having watched competitive players of their own university, but not after having watched competitive players of the other university. Possibly, goal contagion effects are pronounced for socially close others because their goals are more self-relevant. Underlining this assumption, Leander and Shah (2013) revealed that the potential self-relevance of the situational context moderated the goal contagion process. Reading about a friend with a temporally close (vs. distant) deadline made participants work more persistently and perform better. Apparently subjectively close goals may loom larger for oneself (Leander & Shah, 2013), and hence motivation and effort devoted to working toward these goals increase (Peetz et al., 2009).

Another explanation of why people pursue goals of socially close others might be that people ascribe a more positive valence to these goals (Shah, 2003a), which is crucial for goal-directed behavior to occur (e.g. Custers & Aarts, 2005). The importance of a certain goal to significant others informs the person about the subjective value of goal attainment (Moretti & Higgins, 1999; Shah, 2003a). For example, merely thinking about significant others will implicitly prime goals that we think these others want us to pursue (e.g. Fitzsimons & Bargh, 2003; Shah, 2003a, 2003b). In one study, for instance, participants’ ratings on an anagram task of the importance of their performance for a significant other predicted their own anagram persistence and performance, especially when the participants were primed with the name of the significant other (Shah, 2003a). Shah (2003a) argued that this effect depends on the perceived closeness to the significant other. Indeed, the greater participants’ perceived closeness to a friend, the more likely priming with the friend’s named increased the commitment to goals associated with the friend. In our study, the other person was not a significant other, but still the psychological closeness to this person moderated goal pursuit. Feelings of closeness might increase associations between oneself and the other and the positive value of the other’s goal for oneself (Shah, 2003a).

Interestingly, our findings as well as the findings of Loersch et al. (2008) are in line with research on other social contagion effects, such as behavioral and emotional mimicry. In the case of the former, people unconsciously imitated the face-touching behavior of a confederate belonging to their ingroup (i.e. being Christian) to a greater extent than for a confederate belonging to their outgroup (Yabar, Johnston, Miles, & Peace, 2006). In the case of emotional mimicry, negative emotions such as anger or sadness are typically mimicked more for ingroup than outgroup members, as was found by Bourgeois and Hess (2008) and Van Der Schalk et al. (2011). Overall, mimicry both signals and increases affiliation with the interaction partner, like a “social glue” (Lakin, Jefferis, Cheng, & Chartrand, 2003). As goal contagion effects also seem to be stronger between ingroup members and with increased psychological proximity (as shown in the present Study 2), a similar conclusion can be drawn for goal contagion: shared goals may also signal and increase affiliation with interaction partners. Any social contagion effect can only be understood in the specific interpersonal context in which it occurs (Hess & Fischer, 2013).

In contrast to mimicry, however, goal contagion is considered a more “high-level,” powerful form of contagion, because individuals are flexible in choosing the behavioral means to pursue the adopted goal (Aarts et al., 2004; Loersch et al., 2008). If this assumption is true, at first glance our results seem to contradict recent evidence that psychological distance reduces imitation of goals (Genschow, Hansen, Wänke, & Trope, 2016). In one study, participants watched a model pressing one of two keys on the keyboard with either the left or right hand across several trials (Genschow et al., 2016). When participants were asked to imitate the observed behavior they made more errors in pressing the correct key when the observed behavior was presented spatially near than when it was presented spatially distant, indicating that people are better able to focus on goals when imitating distant (compared to proximal) actions. Genschow and Hansen’s paradigm, however, differed from our procedure in two aspects that are theoretically important: First, in their paradigm participants were explicitly instructed to imitate the model’s actions. In our studies, the description of the model’s actions was presented as a task unrelated to the academic performance test. Second, in Genschow et al.’s study the goal of the respective action (i.e. the key) was clearly perceivable, whereas in our studies participants had to infer the goal underlying the model’s actions. Psychological distance may help people focus on a clearly visible goal when consciously trying to imitate the goal (i.e. concentrate on the central aspect of the situation; Trope & Liberman, 2010). In our study, in contrast, the goal was not made explicit. That is, participants observed only goal-related behaviors without a distinct goal. Additionally, participants were not explicitly asked to imitate. In such a situation, psychological distance may cause participants to construe the situation rather broadly, without a focus on specific behaviors but with a wider scope instead (Förster, Liberman, & Kuschel, 2008). With such a broad perspective on the model, imitation of individual actions and contagion with underlying goals may be less likely.

### Limitations

Although we tried to anticipate possible difficulties in our experimental procedure in the registered method, some constraints arose during the process of data collection. In all of our studies, we had a quite unexpected high rate of suspicious participants. After the first pilot study, we changed the cover story slightly and focused on recruiting students from outside the psychology department. Still, there were more suspicious participants than we expected and more than commonly reported in the priming literature. Typically, it is assumed that goal pursuit can be enacted entirely outside of conscious awareness (Bargh, 1990; Bargh & Chartrand, 1999). Our rate of suspicions raises the question if priming effects are really as “automatic” or unconscious as commonly assumed. Future research might investigate if there is instead a “continuum of consciousness” in the process of priming. On the other hand, the high rate of suspicions might have been due to the written funneled debriefing procedure that gave participants too much time to think about the experiments’ purpose. Actually, many participants wrote that they only thought about the connectedness of the study parts once we asked them. During the experimental procedure, they were not necessarily aware of this influence.

## Conclusion

In the present research we investigated the influence of level of construal and psychological distance on goal contagion. We found (initial) evidence that goal contagion effects are not enhanced by a high level of construal but rather by the motivational relevance triggered by psychological closeness. Relevance supposedly enhances the process of “catching” the other’s goal and pursuing it oneself.

## Appendix A. Materials of Pilot Studies 1 and 2

Appendix A1. Funneled debriefing

1. Was glauben Sie, was wir in dieser Studie untersuchen wollen?

**(What do you think we wanted to test with this study?)**

_____________________________________________________________________________

_____________________________________________________________________________

_____________________________________________________________________________

_____________________________________________________________________________

(2) Ist Ihnen irgendein Teil dieser Studie komisch vorgekommen?

O Nein O Ja

**(Did any part of this study seem unusual to you? No/Yes)**

Falls ja, welcher und warum?

**(If yes, which one and why?)**

___________________________________________________________________

___________________________________________________________________

(3) Glauben Sie, dass der erste Teil der Studie „Andere Menschen kennen lernen“ mit dem zweiten Computer-Teil der Studie „Entscheidungsfindung” zusammenhing?

O NeinO Ja

**(Do you think that the first part of the study “getting to know other people” was related to the second computer part “decision making”? No/Yes)**

Falls ja, wie genau?

**(If yes, how exactly?)**

___________________________________________________________________

___________________________________________________________________

___________________________________________________________________

(4) Glauben Sie, dass der erste Teil der Studie „Andere Menschen kennen lernen“ ihre Antworten im zweiten Computer-Teil der Studie „Entscheidungsfindung” beeinflusst hat?

**(Do you think that the first part of the study “getting to know other people” influenced your behavior or answers during the second computer part “decision making”? No/Yes)**

O NeinO Ja

Falls ja, was genau?

**(If yes, how exactly?)**

___________________________________________________________________

___________________________________________________________________

___________________________________________________________________

(5) Was glauben Sie, was wir in dieser Studie untersuchen wollen?

**(What do you think we wanted to test with this study?)**

____________________________________________________________________________

____________________________________________________________________________

____________________________________________________________________________

____________________________________________________________________________

Appendix A2. German versions of the vignettes

*Academic goal*

“Die Studentin Anita ist wirklich zufrieden mit ihrem Studium und ihrem Universitätsleben. Beispielsweise besucht sie Vorlesungen und trifft sich mit Mitstudenten um gemeinsam zu lernen. Sie sitzt häufig in der Bibliothek um Lehrbücher und Artikel zu lesen und um Zusammenfassungen zu schreiben.”

*Social goal*

“Die Studentin Anita ist wirklich zufrieden mit ihrem Studium und ihrem Universitätsleben. Beispielsweise besucht sie Sportkurse und trifft sich mit Freunden um zu quatschen. Häufig sitzt sie in einem Café um Zeitschriften und Updates auf Sozialen Medien zu lesen und Kommentare zu den Neuigkeiten ihrer Freunde zu schreiben.”

Appendix A3. Multiple choice questions about vignettes in Pilot Study 1

**1. Was macht Anita regelmäßig? (What does Anita do regularly?)**

- Sie geht in Sportkurse. (She goes to sports classes.)
- Sie arbeitet in einem Restaurant. (She works in a restaurant.)
- Sie geht in die Vorlesungen. (She goes to lectures.)

**2. Was macht Anita gerne? (What does Anita like to do?)**

- Mit Kommilitonen lernen (Study with fellow students)
- Mit Freunden quatschen (Chat with friends)
- Lange im Wald spazieren gehen (Go for long walks in the woods)

**3. Was liest Anita normalerweise und wo? (What does Anita usually read and where?)**

- Zeitungen im Zug (Newspapers on the train)
- Lehrbücher in der Bibliothek (textbooks in a library)
- Updates auf sozialen Netzwerken in einem Café (social media updates in a café)

Appendix A4. Words used for the word categorization task (adopted from Leander & Shah, 2013)

*Academic goal*

- akzeptieren (accept)
- verdienen (earn)
- erreichen (attain)
- Mühe (effort)
- Stress (stress)
- Problem (problem)
- Gelingen (succeed)
- Fragen (question)
- Erfüllen (fulfill)
- Abschluss (graduation)

*Social goal*

- Respekt (respect)
- angeben (boast)
- anbieten (offer)
- kooperieren (cooperate)
- antworten (answer)
- Lob (praise)
- erklären (instruct)
- Mittagessen (luncheon)
- beliebt (popular)
- zufriedenstellen (satisfy)

*Ambiguous*

- teilnehmen (attend)
- sich bekannt machen (acquaint)
- Zustimmung (approval)
- Treffen (meeting)
- Diskussion (discussion)
- empfehlen (commendation)
- ehren (honor)
- Bekanntheitsgrad (notoriety)
- Nachricht versenden (e-mail)
- Interesse (interest)

Appendix A5. German versions of the extended vignettes

*Academic*

“Die Studentin Anita ist wirklich zufrieden mit ihrem Studium und ihrem Universitätsleben. Beispielsweise besucht sie Vorlesungen und macht sich währenddessen Notizen zu den Inhalten. Auch trifft sie sich mit Mitstudenten in der Mensa um gemeinsam Prüfungsstoff zu lernen und zu diskutieren. Anita sitzt häufig in der Bibliothek um Lehrbücher und Artikel zu lesen und um Zusammenfassungen zu schreiben. Sie informiert sich über Vorträge und Diskussionen, die in ihrer Umgebung stattfinden, und besucht diese gerne. In ihrem Studentenheim-Zimmer hat Anita eine Sammlung von Fachzeitschriften in ihrem Regal stehen und sie tauscht sich mit ihren Mitbewohnern über die Themen ihres Studiums aus …”

*Social*

“Die Studentin Anita ist wirklich zufrieden mit ihrem Studium und ihrem Universitätsleben. Beispielsweise besucht sie Sportkurse und trifft sich danach mit Freundinnen zum Quatschen. Auch trifft sie sich mit Mitstudenten in der Mensa um gemeinsam Kaffee zu trinken und sich auszutauschen. Anita sitzt häufig sitzt in einem Café um Zeitschriften und Updates auf Sozialen Medien zu lesen und um Kommentare zu den Neuigkeiten ihrer Freunde zu schreiben. Sie informiert sich über Partys und Flohmärkte, die in ihrer Umgebung stattfinden, und besucht diese gerne. In ihrem WG-Zimmer hat Anita eine Sammlung von Pop-CDs in ihrem Regal stehen und sie tauscht sich mit ihren Mitbewohnern über die Neuigkeiten aus dem Freundeskreis aus …”

Appendix A6. Words used for the lexical decision task

*Academic words (and nonwords used with same length)*

- Mühe (effort) Kalp
- Stress (stress) Kaling
- erreichen (attain) bullwaren
- gelingen (succeed) huptewen
- Abschluss (graduation) Attupowen
- Referat (presentation) Datukal
- Lehrveranstaltung (course) Entepolitowierung
- anmelden (register) daringen
- Seminararbeit (term paper) Fringullengen
- Curriculum (curriculum) Gruntepede
- Stundenplan (schedule) Humtefeller
- Professor (professor) Introkept
- absolvieren (complete) jurenalagen
- leisten (perform) karopen
- Theorie (theory) Mitwane
- bestehen (pass) nuraplen
- Wissenschaft (science) Ottwagenhatt
- Stipendium (scholarship) Prupläbium
- Erfolg (success) Renzo
- antreben (pursuit) rindven

*Social words (and nonwords used with same length)*

- lachen (laugh) suklen
- Gesellschaft (company) Tramelkenaft
- Freude (joy) Urenta
- Ausgehen (go out) vengeten
- Einladung (invitation) Wrunkmung
- Wochenende (weekend) Lintewuper
- spielen (play) anrowen
- Gefühle (feelings) Ballule
- verbinden (connect) drinteren
- Verabredung (date) Ennelierung
- shoppen (shopping) frinden
- Hobbies (hobbies) Grendus
- Kino (cinema) Haru
- Beziehung (relationship) Immentiel
- abhängen (hang out) jammuren
- tanzen (dance) hazzen
- Gemeinschaft (community) Kupplenarung
- Vertrauen (trust) Linfrunge
- Anschluss (connection) Minrocken
- Familie (family) Norites

*Neutral words (and nonwords used with same length)*

- putzen (clean) oreten
- Auto (car) Püpf
- zeichnen (draw) rettaren
- Besorgungen (errands) Sruggelegen
- Post (mail) Terp
- fernsehen (watching TV) untawegen
- abholen (fetch) kongwen
- fotografieren (take picture) huffelipenden
- dekorieren (decorate) denketoden
- umziehen (move) verguwen
- Computer (computer) Hammutel
- Haustürschlüssel (latchkey) Gemmeltarentopen
- Krankheit (disease) Frankelte
- Lebensmittel (grocery) Enjommeltent
- Haustier (pet) Drunnpel
- Sammlung (collection) Barukkel
- Kleidung (clothes) Antropem
- Mülltonne (dustbin) Uratunnen
- Garten (garden) Winjur
- Badezimmer (bathroom) Verottewen

## Appendix B. Materials of Studies 1 and 2

Appendix B1. Studies 1 and 2: funneled debriefing

1. Was glauben Sie, was wir in dieser Studie untersuchen wollen?

**(What do you think we wanted to test with this study?)**

____________________________________________________________________________

____________________________________________________________________________

(2) Ist Ihnen irgendein Teil dieser Studie komisch vorgekommen?

NeinJa

**(Did any part of this study seem unusual to you? No/Yes)**

Falls ja, welcher und warum?

**(If yes, which part and why?)**

__________________________________________________________________

__________________________________________________________________

__________________________________________________________________

(3) Glauben Sie, dass einer der beiden ersten Teile der Studie, die “Vorstellungsaufgabe” bzw. “Andere Menschen kennen lernen” (Studie 2: “verbales Beschreibungsvermögen”) mit dem zweiten Teil der Studie “Akademischer Leistungstest” zusammenhing?

NeinJa

**(Do you think that one of the first parts of the study, “imagination task” or “getting to know other people”(Study 2: “verbal description task”), was related to the second part, “academic performance test”? No/Yes)**

Falls ja, wie genau?

**(If yes, how exactly?)**

_________________________________________________________________

_________________________________________________________________

_________________________________________________________________

(4) Glauben Sie, dass einer der beiden ersten Teile der Studie, die „Vorstellungsaufgabe“ bzw. “Andere Menschen kennen lernen” (Studie 2: “verbales Beschreibungsvermögen”) ihre Antworten im zweiten Teil der Studie “Akademischer Leistungstest” beeinflusst hat?

**(Do you think that one of the first parts of the study, “imagination task” or “getting to know other people” (Study 2: “verbal description task”) influenced your behavior or answers during the second part, “academic performance test”? No/Yes)**

NeinJa

Falls ja, was genau?

**(If yes, how exactly?)**

__________________________________________________________________

__________________________________________________________________

__________________________________________________________________

Appendix B2. Study 1: “How” priming condition

Appendix B3. Study 1: “Why” priming condition

Appendix B4. Studies 1 and 2: anagrams

**Table:**

No.	Letters	Possible solutions
1	ITSEL	Liste, Stiel, Stile, eilst, liest, seilt, steil, teils
2	NREGE	Genre, Regen, enger, gerne, regne
3	HESAN	Hasen, sahen, Sahne, Hanse, nahes
4	NSEIL	Insel, Linse, Niels, senil, Lenis
5	EIREN	Eiern, Irene, Niere, einer, reine
6	NGLAE	Angel, Nagel, Algen, nagle, angle, lagen, lange, Galen

## References

[CIT0001] Aarts H., Chartrand T. L., Custers R., Danner U., Dik G., Jefferis V. E., & Cheng C. M. (2005). Social stereotypes and automatic goal pursuit. *Social Cognition*, 23, 465–490. doi:10.1521/soco.2005.23.6.465

[CIT0002] Aarts H., Dijksterhuis A., & Dik G. (2008). Goal contagion: Inferring goals from others’ actions–and what it leads to In Shah J. Y. & Gardner W. L. (Eds.), *Handbook of motivation science* (pp. 265–280). New York, NY: The Guilford Press.

[CIT0003] Aarts H., Gollwitzer P. M., & Hassin R. R. (2004). Goal contagion: Perceiving is for pursuing. *Journal of Personality and Social Psychology*, 87, 23–37. doi:10.1037/0022-3514.87.1.23 15250790

[CIT0004] Alter A. L., Oppenheimer D. M., & Zemla J. C. (2010). Missing the trees for the forest: A construal level account of the illusion of explanatory depth. *Journal of Personality and Social Psychology*, 99, 436–451. doi:10.1037/a0020218 20658836

[CIT0005] Bargh J. A. (1990). Goal ≠ intent: Goal-directed thought and behavior are often unintentional. *Psychological Inquiry*, 1, 248–251. doi:10.1207/s15327965pli0103_14

[CIT0006] Bargh J. A., Chaiken S., Govender R., & Pratto F. (1992). The generality of the automatic attitude activation effect. *Journal of Personality and Social Psychology*, 62, 893–912. doi:10.1037/0022-3514.62.6.893 1619549

[CIT0007] Bargh J. A., & Chartrand T. L. (1999). The unbearable automaticity of being. *American Psychologist*, 54, 462–479. doi:10.1037/0003-066X.54.7.462

[CIT0008] Bargh J. A., & Chartrand T. L. (2000). The mind in the middle: A practical guide to priming and automaticity research In Reis H. T. & Judd C. M. (Eds.), *Handbook of research methods in social and personality psychology* (pp. 253–285). New York, NY: Cambridge University Press.

[CIT0009] Bargh J. A., Gollwitzer P. M., Lee-Chai A., Barndollar K., & Trötschel R. (2001). The automated will: Nonconscious activation and pursuit of behavioral goals. *Journal of Personality and Social Psychology*, 81, 1014–1027. doi:10.1037/0022-3514.81.6.1014 11761304 PMC3005626

[CIT0010] Bourgeois P., & Hess U. (2008). The impact of social context on mimicry. *Biological Psychology*, 77, 343–352. doi:10.1016/j.biopsycho.2007.11.008 18164534

[CIT0011] Chartrand T. L., & Bargh J. A. (1996). Automatic activation of impression formation and memorization goals: Nonconscious goal priming reproduces effects of explicit task instructions. *Journal of Personality and Social Psychology*, 71, 464–478. doi:10.1037/0022-3514.71.3.464

[CIT0012] Custers R., & Aarts H. (2005). Beyond priming effects: The role of positive affect and discrepancies in implicit processes of motivation and goal pursuit. *European Review of Social Psychology*, 16, 257–300. doi:10.1080/10463280500435919

[CIT0013] Custers R., & Aarts H. (2010). The unconscious will: How the pursuit of goals operates outside of conscious awareness. *Science*, 329, 47–50. doi:10.1126/science.1188595 20595607

[CIT0014] Dik G., & Aarts H. (2007). Behavioral cues to others’ motivation and goal pursuits: The perception of effort facilitates goal inference and contagion. *Journal of Experimental Social Psychology*, 43, 727–737. doi:10.1016/j.jesp.2006.09.002

[CIT0015] Faul F., Erdfelder E., Lang A.-G., & Buchner A. (2007). G* Power 3: A flexible statistical power analysis program for the social, behavioral, and biomedical sciences. *Behavior Research Methods*, 39, 175–191. doi:10.3758/BF03193146 17695343

[CIT0016] Fazio R. H. (1990). A practical guide to the use of response latency in social psychological research In Hendrick C. & Clark M. S. (Eds.), *Research methods in social and personality psychology* (pp. 74–97). Thousand Oaks, CA, US: Sage Publications, Inc.

[CIT0017] Fitzsimons G. M., & Bargh J. A. (2003). Thinking of you: Nonconscious pursuit of interpersonal goals associated with relationship partners. *Journal of Personality and Social Psychology*, 84, 148–164. doi:10.1037/0022-3514.84.1.148 12518976 PMC3011819

[CIT0018] Fitzsimons G. M., & Fishbach A. (2010). Shifting closeness: Interpersonal effects of personal goal progress. *Journal of Personality and Social Psychology*, 98, 535–549. doi:10.1037/a0018581 20307127

[CIT0019] Förster J., Liberman N., & Kuschel S. (2008). The effect of global versus local processing styles on assimilation versus contrast in social judgment. *Journal of Personality and Social Psychology*, 94, 579–599. doi:10.1037/0022-3514.94.4.579 18361673

[CIT0020] Freitas A. L., Gollwitzer P., & Trope Y. (2004). The influence of abstract and concrete mindsets on anticipating and guiding others’ self-regulatory efforts. *Journal of Experimental Social Psychology*, 40, 739–752. doi:10.1016/j.jesp.2004.04.003

[CIT0021] Fujita K., Henderson M. D., Eng J., Trope Y., & Liberman N. (2006). Spatial distance and mental construal of social events. *Psychological Science*, 17, 278–282. doi:10.1111/j.1467-9280.2006.01698.x 16623682

[CIT0022] Fujita K., Trope Y., Liberman N., & Levin-Sagi M. (2006). Construal levels and self-control. *Journal of Personality and Social Psychology*, 90, 351–367. doi:10.1037/0022-3514.90.3.351 16594824 PMC3153425

[CIT0023] Genschow O., Hansen J., Wänke M., & Trope Y. (2016). *Psychological distance modulates goal-based versus movement-based imitation*. Unpublished manuscript University of Ghent and University of Salzburg. 10.1037/xhp0000654 31135170

[CIT0024] Hamilton D. L., Katz L. B., & Leirer V. O. (1980). Cognitive representation of personality impressions: Organizational processes in first impression formation. *Journal of Personality and Social Psychology*, 39, 1050–1063. doi:10.1037/h0077711

[CIT0025] Hansen J., Alves H., & Trope Y. (2016). Psychological distance reduces literal imitation: Evidence from an imitation-learning paradigm. *Journal of Experimental Psychology: Human Perception and Performance*, 42, 320–330. doi:10.1037/xhp0000150 26414166

[CIT0026] Hansen J., & Trope Y. (2013). When time flies: How abstract and concrete mental construal affect the perception of time. *Journal of Experimental Psychology: General*, 142, 336–347. doi:10.1037/a0029283 22800441

[CIT0027] Hassin R. R., Aarts H., & Ferguson M. J. (2005). Automatic goal inferences. *Journal of Experimental Social Psychology*, 41, 129–140. doi:10.1016/j.jesp.2004.06.008

[CIT0028] Hassin R. R., Bargh J. A., & Uleman J. S. (2002). Spontaneous causal inferences. *Journal of Experimental Social Psychology*, 38, 515–522. doi:10.1016/S0022-1031(02)00016-1

[CIT0029] Heider F., & Simmel M. (1944). An experimental study of apparent behavior. *The American Journal of Psychology*, 57, 243–259. doi:10.2307/1416950

[CIT0030] Hess U., & Fischer A. (2013). Emotional mimicry as social regulation. *Personality and Social Psychology Review*, 17(2), 142–157. doi:10.1177/1088868312472607 23348982

[CIT0031] Lakens D. (2013). Calculating and reporting effect sizes to facilitate cumulative science: A practical primer for t-tests and ANOVAs. *Frontiers in Psychology*, 4 10.3389/fpsyg.2013.00863 10.3389/fpsyg.2013.00863 PMC3840331 24324449

[CIT0032] Lakin J. L., Jefferis V. E., Cheng C. M., & Chartrand T. L. (2003). The chameleon effect as social glue: Evidence for the evolutionary significance of nonconscious mimicry. *Journal of Nonverbal Behavior*, 27, 145–162. doi:10.1023/A:1025389814290

[CIT0033] Leander N. P., & Shah J. Y. (2013). For whom the goals loom: Context-driven goal contagion. *Social Cognition*, 31, 187–200. doi:10.1521/soco.2013.31.2.187

[CIT0034] Leander N. P., Shah J. Y., & Chartrand T. L. (2011). The object of my protection: Shielding fundamental motives from the implicit motivational influence of others. *Journal of Experimental Social Psychology*, 47, 1078–1087. doi:10.1016/j.jesp.2011.04.016

[CIT0035] Liberman N., Förster J., & Higgins E. T. (2007). Completed vs. interrupted priming: Reduced accessibility from post-fulfillment inhibition. *Journal of Experimental Social Psychology*, 43, 258–264. doi:10.1016/j.jesp.2006.01.006

[CIT0036] Liberman N., & Trope Y. (1998). The role of feasibility and desirability considerations in near and distant future decisions: A test of temporal construal theory. *Journal of Personality and Social Psychology*, 75, 5–18. doi:10.1037/0022-3514.75.1.5

[CIT0037] Loersch C., Aarts H., Payne B. K., & Jefferis V. E. (2008). The influence of social groups on goal contagion. *Journal of Experimental Social Psychology*, 44, 1555–1558. doi:10.1016/j.jesp.2008.07.009

[CIT0038] Long D. L., & Golding J. M. (1993). Superordinate goal inferences: Are they automatically generated during comprehension? *Discourse Processes*, 16(1–2), 55–73. doi:10.1080/01638539309544829

[CIT0039] Moretti M. M., & Higgins E. T. (1999). Internal representations of others in self-regulation: A new look at a classic issue. *Social Cognition*, 17, 186–208. doi:10.1521/soco.1999.17.2.186

[CIT0040] Peetz J., Wilson A. E., & Strahan E. J. (2009). So far away: The role of subjective temporal distance to future goals in motivation and behavior. *Social Cognition*, 27, 475–495. doi:10.1521/soco.2009.27.4.475

[CIT0041] Scholl B. J., & Tremoulet P. D. (2000). Perceptual causality and animacy. *Trends in Cognitive Sciences*, 4(8), 299–310. doi:10.1016/S1364-6613(00)01506-0 10904254

[CIT0042] Semin G. R., & Fiedler K. (1988). The cognitive functions of linguistic categories in describing persons: Social cognition and language. *Journal of Personality and Social Psychology*, 54, 558–568. doi:10.1037/0022-3514.54.4.558

[CIT0043] Semin G. R., & Fiedler K. (1991). The linguistic category model, its bases, applications and range. *European Review of Social Psychology*, 2, 1–30. doi:10.1080/14792779143000006

[CIT0044] Shah J. (2003a). Automatic for the people: How representations of significant others implicitly affect goal pursuit. *Journal of Personality and Social Psychology*, 84, 661–681. doi:10.1037/0022-3514.84.4.661 12703642

[CIT0045] Shah J. (2003b). The motivational looking glass: How significant others implicitly affect goal appraisals. *Journal of Personality and Social Psychology*, 85, 424–439. doi:10.1037/0022-3514.85.3.424 14498780

[CIT0046] Soderberg C. K., Callahan S. P., Kochersberger A. O., Amit E., & Ledgerwood A. (2015). The effects of psychological distance on abstraction: Two meta-analyses. *Psychological Bulletin*, 141, 528–548. doi:10.1037/bul0000005 25420220

[CIT0047] Tabachnick B. G., & Fidell L. S. (2013). *Using multivariate statistics* (6th ed.). Boston: Pearson Education.

[CIT0048] Trope Y., & Liberman N. (2010). Construal-level theory of psychological distance. *Psychological Review*, 117, 440–463. doi:10.1037/a0018963 20438233 PMC3152826

[CIT0049] Uleman J. S., Newman L. S., & Moskowitz G. B. (1996). People as flexible interpreters: evidence and issues from spontaneous trait inference In Zanna M. P. (Ed.), *Advances in experimental social psychology* (Vol. 28, pp. 211–279). San Diego, CA: Academic Press.

[CIT0050] Vallacher R. R., & Wegner D. M. (1987). What do people think they’re doing? Action identification and human behavior. *Handbook of Motivation and Cognition: Foundations of Social Behavior*, 94, 3–15. http://dx.doi.org/10.1037/0033-295X.94.1.3

[CIT0051] Van Der Schalk J., Fischer A., Doosje B., Wigboldus D., Hawk S., Rotteveel M., & Hess U. (2011). Convergent and divergent responses to emotional displays of ingroup and outgroup. *Emotion*, 11, 286–298. doi:10.1037/a0022582 21500898

[CIT0052] Wegner D. M., & Vallacher R. R. (1986). Action identification In Sorrentino R. M. & Higgins E. T. (Eds.), *Handbook of motivation and cognition: Foundations of social behavior* (pp. 550–582). New York, NY: Guilford.

[CIT0053] Weingarten E., Chen Q., McAdams M., Yi J., Hepler J., & Albarracín D. (2016). From primed concepts to action: A meta-analysis of the behavioral effects of incidentally presented words. *Psychological Bulletin*, 142, 472–497. doi:10.1037/bul0000030 26689090 PMC5783538

[CIT0054] Yabar Y., Johnston L., Miles L., & Peace V. (2006). Implicit behavioral mimicry: Investigating the impact of group membership. *Journal of Nonverbal Behavior*, 30, 97–113. doi:10.1007/s10919-006-0010-6

